# Developing as health professionals through community volunteering: exploring the value of a partnership between medical students and primary schools online compared to in-person

**DOI:** 10.1186/s12909-023-04032-7

**Published:** 2023-01-24

**Authors:** Alexandra M. Cardoso Pinto, Sajan B. Patel, Morwenna Stephens, Payal Guha, Ana Baptista, Susan Smith

**Affiliations:** 1 School of Medicine, Imperial College London, London, UK; 2 Medical Education Research Unit, Faculty of Medicine, Imperial College London, London, UK

**Keywords:** Student society, Volunteering, Primary education, Lockdown, Medical students

## Abstract

**Introduction:**

Imperial College Teddy Bear Hospital (ICSM-TBH) is a student-led volunteering group, which uses interactive, play-based teaching to educate school pupils aged 5–7 years about healthy lifestyles and healthcare. During the COVID-19 pandemic, volunteering sessions shifted online. The aim of this study was to compare the value of online and in-person ICSM-TBH volunteering for volunteers and school pupils.

**Methods:**

Undergraduate university students at Imperial College London (medicine can be taken as a first degree in the UK) who volunteered with ICSM-TBH between 2019 and 22 were invited to complete an anonymous online questionnaire evaluating their experiences of volunteering online and in-person through Likert-scale questions. Those who completed the questionnaire were also invited to an interview. Teachers who hosted online ICSM-TBH sessions were also invited to an in-person interview, exploring their view of their pupils’ experiences with these sessions. Questionnaire results were analysed through descriptive statistics. Interviews were analysed through inductive thematic analysis.

**Results:**

Thirty-two university students completed the questionnaire. Of these, 9 experienced both in-person and online volunteering, all of whom preferred in-person volunteering. For those who only volunteered in-person, 92% reported that ICSM-TBH sessions were a positive experience, compared to 100% who volunteered online; 92% in person volunteers agreed or strongly agreed that ICSM-TBH volunteering in person improved their mood, compared to 89% online; and 100% agreed or strongly agreed that ICSM-TBH volunteering in person helped them feel part of a community, compared to 84% online. A total of 12 volunteers and 4 teachers were interviewed, from whom five themes emerged: interaction and engagement (interaction and engagement between pupils and volunteers was more readily achieved in-person); personal and professional development (both online and in-person sessions enabled volunteers to gain valuable skills); community and social (greater sense of community was established in-person); emotional wellbeing and enjoyment (both modalities were enjoyed by volunteers and pupils); and workload (online sessions were more convenient for volunteers but with risk of screen fatigue).

**Conclusion:**

Overall, both in-person and online volunteering were of substantial benefit to volunteers and school pupils. However, most teachers and volunteers preferred in-person volunteering.

## Introduction

Teddy Bear Hospital (TBH) is a university student-led volunteering group present in many universities and medical schools internationally [1–5]. It utilises play to promote healthy living among primary school children (aged 5 to 7 years) and reduce their anxiety surrounding hospitals and healthcare professionals [6]. Imperial College London School of Medicine Teddy Bear Hospital (ICSM-TBH) has run since 2014 with over 30 volunteers annually attending primary schools in West London and providing play-based small-group teaching.

Prior to the COVID-19 pandemic, ICSM-TBH utilised in-person interactive small group stations (with five pupils taught by one volunteer) to cover topics such as nutrition, the human body, surgery, and emergencies. The volunteer is provided with a guide on the station and equipment, for example, a ‘surgery bear’ with removable organs and toy medical equipment for the surgery station. Each station runs for 7 minutes before the pupils rotate to the next station. The session ends in a large group, with the pupils answering questions and reporting on what they have learnt. During the pandemic, necessary changes were made for the safety of schools and the volunteers. Interactive presentations were created covering the topics of nutrition, emergencies, hand hygiene, and the brain. These presentations involved questions for the pupils and mini games. The method of virtual presentation depended on the capacity of the school, most were presented by a pair of volunteers to a large group of pupils sat in front of their classroom whiteboard, however, some schools were able to have breakout rooms with each pupil on their own device allowing for the presentation to smaller groups.

The COVID-19 pandemic brought challenges to most community volunteering schemes including ICSM-TBH. Lockdowns prohibited large group gatherings, meaning that in-person school visits were suspended. In response, ICSM-TBH adapted its sessions to an online format, as described above. Despite numerous studies investigating virtual teaching for medical students [7–11] and the impact of medical students volunteering in-person [12–14] in clinical settings, to our knowledge there have been no reports of the impact of virtual community volunteering. Additionally, since the pandemic has led to poorer mental health amongst university students [15], and in-person volunteering improves mental health [16], the impact of this novel online delivery of ICSM-TBH on volunteer experience, wellbeing, and sense of community was examined.

## Aims & objectives

The aim of this study was to understand the value of one form of online community volunteering, ICSM-TBH, for university students compared to in-person equivalents.

Specifically, the objectives included comparing online and in-person:

- Volunteer teaching experiences,
- Volunteer wellbeing and sense of community, and
- Teacher perceptions of children’s learning.

## Methods

A mixed-methods methodology with a focus on qualitative data was adopted, to enable more detailed exploration of participants’ experiences and establish links between findings and practice, as explained below [17].

### Data collection and recruitment

**Fig. 1:**
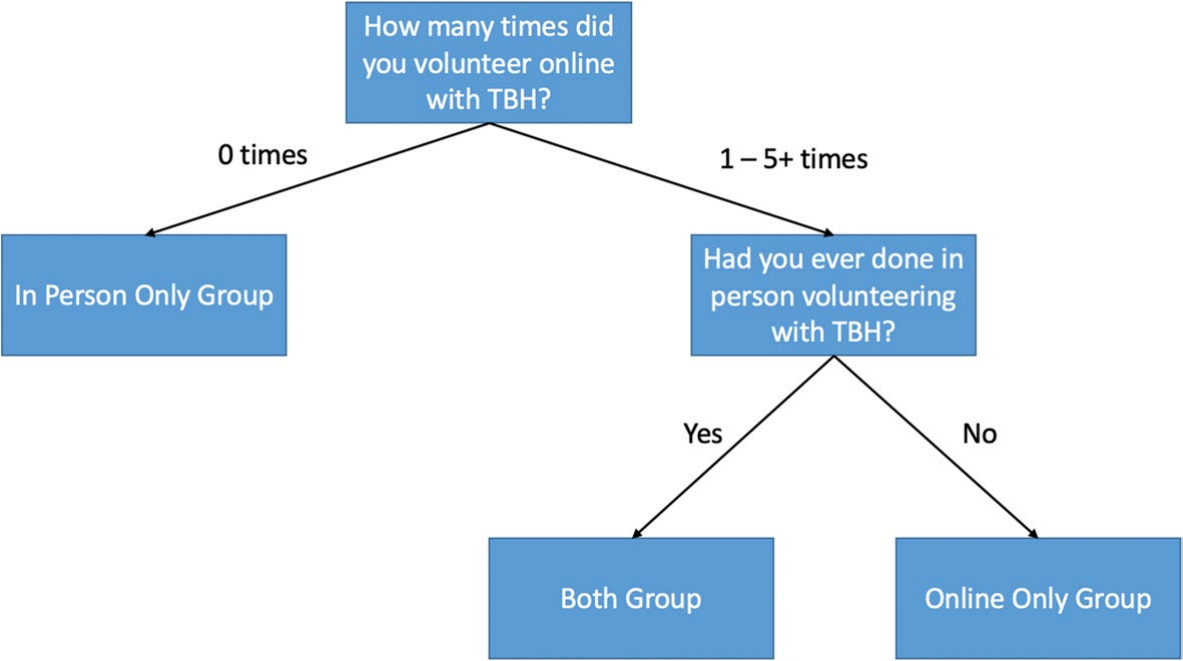
Criteria for Volunteer Groups

ICSM-TBH volunteered in approximately 10 primary schools online and 21 in-person between 2019 and 22. School pupils participated in online sessions either from school, with the session projected on a screen, or from home (schools had already provided pupils with laptops where necessary). A total of 140 students from Imperial College London who had volunteered with ICSM-TBH between 2019 and 2022 were invited to complete an anonymous survey on Qualtrics exploring their volunteering experiences. This was the quantitative component of the mixed-methods approach and enabled a broader pool of participants to provide an overview of their experiences with ICSM-TBH [18]. Participants were recruited through ICSM-TBH social media and weekly newsletter advertisements between October 2021 and March 2022. ICSM-TBH is open to all students at Imperial College. Although almost all volunteers are medical students, students from other courses were eligible to take part. Participants were split into three groups: online volunteers, in-person volunteers and both (Fig. 1). The online and both groups answered questions on their perspectives of online volunteering, and the in-person group answered questions on their perspectives of in-person volunteering.

Participants completing the survey were invited to sign-up to a semi-structured interview further exploring their volunteering experience. These formed the primary qualitative component of the mixed-methods approach, alongside free-text survey questions, and enabled views and experiences to be explored in greater depth from a smaller subset of participants [19]. Such detailed views also directly help inform future practice. Interviews were held online using Microsoft Teams with consent forms provided in advance. Volunteers who completed the interview were given the opportunity to enter a raffle for the chance to win one of seven £10 Amazon vouchers.

Primary school teachers who worked with ICSM-TBH online, which were a total of 12, were invited by email to a semi-structured in-person interview, covering reasons for involvement with ICSM-TBH, their experiences online and in-person, and the challenges and benefits they have witnessed for themselves and their pupils.

All interviews were audio recorded and transcribed verbatim manually; completed transcripts were sent to participants for redaction, then anonymised.

For simplicity, in the present study the term “volunteer” refers to university or medical student involved with ICSM-TBH; “teacher” denotes the primary school educator; “pupil” refers to the primary school child; and “participant” means a volunteer or a teacher who participated in this study.

### Data storage and protection

All data were stored on secure Imperial College London servers. Recordings were deleted upon full transcription. Survey results were stored on Qualtrics.

### Data analysis

Survey Likert-scale questions were analysed descriptively using Microsoft Excel. Interviews and free-text survey results were analysed thematically, following an inductive approach, with coding performed on NVivo 12.0. Braun and Clarke’s stages of thematic analysis were followed as guidance [20]. Each interview was coded by at least two researchers.

### Ethical approval

Ethical approval was granted through the Imperial College London Education Ethics Review Process (EERP2021–107).

### Reflexivity statement

All student authors are former presidents of ICSM-TBH who helped to create and run the online teaching syllabus and therefore may be biased in having positive views of the volunteering. Participants may also know the authors as authority figures within ICSM-TBH, which may have skewed responses to sound more positive. This was mitigated by offering an external interviewer unknown to participants.

## Results

A total of 51 volunteers participated in the survey, however, 19 did not fully complete it so were excluded, giving a total of 32 completed responses. Overall, 10 participants had volunteered for ICSM-TBH online only, 13 had volunteered in-person only and 9 volunteered both online and in-person.

In the academic year 2020/21, the median year of academic studies amongst online volunteer participants was year 1 (range 1–2); the median for participants who volunteered both online and in-person was year 3 (range 2–5); and in-person only volunteer participants was also year 3 (range 2–5).

All volunteer participants who completed the survey, in addition to 12 teachers, were invited to an interview. Overall, 12 volunteer participants (VP) and 4 teacher participants (TP) were interviewed. Of these volunteer participants, 10 were medical students and 2 were life science students; there were no difference in results between these groups, apart from relation to clinical practice. Of the 12 volunteer participants, 5 had participated in both online and in-person ICSM-TBH sessions, 3 had only volunteered in-person with ICSM-TBH and 4 had only volunteered online with ICSM-TBH. However, apart from one volunteer who had only volunteered in-person, all other participants interviewed had experience of both in-person and online volunteering through other organisations.

### Survey results

**Fig. 2:**
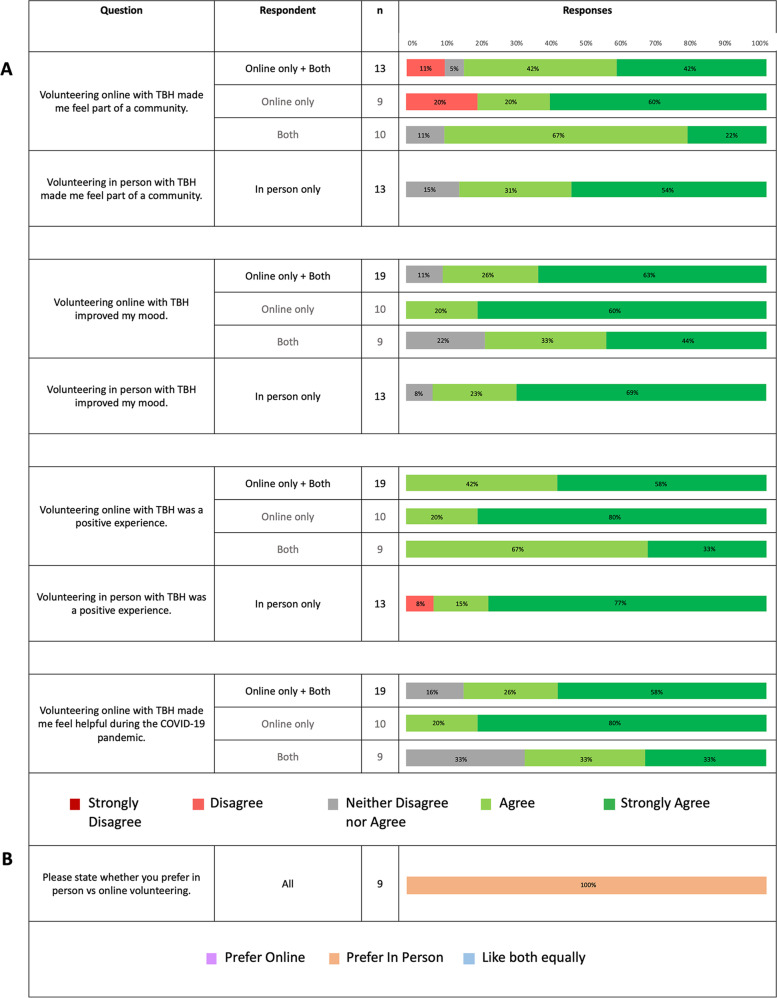
Survey Results (**A**) Volunteer experiences of online and in-person volunteering (**B**) Volunteer preferences between online and in-person volunteering

For those who only volunteered in-person, 92% reported that ICSM-TBH sessions were a positive experience, compared to 100% who volunteered online (online only volunteers and both). 92% in-person volunteers agreed or strongly agreed that ICSM-TBH volunteering in-person improved their mood, compared to 89% online; and 100% agreed or strongly agreed that ICSM-TBH volunteering in-person helped them feel part of a community, compared to 84% online (Fig. 2). Of those volunteer participants who volunteered both online and in-person with ICSM-TBH, all preferred in-person (Fig. 2).

### Thematic analysis

Five primary themes emerged from the interviews:

(i) Interaction and Engagement: volunteers’ experiences interacting with and engaging the school pupils across both modalities.
(ii) Personal and Professional Development: the various skills and lessons learnt by volunteers both in their personal and professional lives and how these will be used in future.
(iii) Community and Social: volunteers’ perspectives around the sense of community and social aspect across both modalities.
(iv) Emotional Wellbeing and Enjoyment: volunteers’ experiences of their emotional wellbeing and their enjoyment from the volunteering across both modalities.
(v) Workload: academic workload of medical students and how this impacted volunteering across both modalities.

(i). *Interaction and Engagement*

In-person volunteering allowed for greater interaction and engagement from pupils. One explanation was the ability to capture non-visual cues in-person.

*“There are little cues that we don’t necessarily catch online (…) [and] body language is not fully there” (VP6).*

Visual cues are crucial enabling effective teamwork, making “*co-running the station with someone in-person a lot easier*” (VP10) and facilitates awareness of the pupil’s engagement levels.

*“If a certain kid isn’t being as engaged in the activity as others, it’s easier to pick up on that [in-person] (...)* *and tailor how you’re getting your message across” (VP8).*

The lack of physical presence and interaction online made volunteers feel sessions were impersonal, which discouraged some from participating.

Volunteer participants also reported difficulty focusing online, for example,

*“You can get a bit distracted (…) all my emails and everything keep tabbing through” (VP1).*

Technical challenges, including WiFi or the use of software for teaching, disrupted the flow of sessions.

*“Wondering if they can hear you and you can’t hear them half the time. So you just kind of have to fake, (…) “yeah, I think I heard the right answer!” (VP10).*

The nature of online sessions made it more difficult to individualise teaching methods for pupils and was further challenged by greater numbers of pupils taught at once.

*“(…) you couldn’t speak to children individually. So you didn’t get that like one-to- one” (VP9).*

Technical and communication challenges of online volunteering also demanded substantially more input from teachers than in-person volunteering. Whilst this removed the volunteers’ challenging responsibility to keep pupil’s attention, it made interactions with pupils indirect.

*“At some points we’re just interacting with the teacher instead of the kids. Because like we say something that kids can’t grab, and the teacher has to repeat it and then the kids respond to the teacher” (VP2)*

(ii). *Personal and Professional Development*

Participants valued ICSM-TBH as a space for personal and professional growth, including skill development. Confidence was commonly mentioned; volunteer participants felt more confident engaging with children following in-person sessions.

*“I was initially quite scared of children and didn’t really know how to approach and speak to children and how to deal with certain situations regarding children, [following in-person volunteering] I think [I am] definitely currently a lot more comfortable with that, [and have] step[ed] out of my comfort zone.” (VP4).*

Some volunteer participants noted that the online modality made them *“more shy”* (VP9) whereas others felt that the being in the comfort of your own home provided a sense of familiarity making it *“less intimidating.”* (VP7).

Communication skills are also crucial in the effective delivery of volunteering sessions. In-person sessions were felt to offer a space to develop these. Particularly gaining experience *“communicating medical topics to children”* (VP8), and with peers. Volunteer participants noted a difference in communication skills required for online delivery versus in-person.

*“You have to change (…) how you communicate over a screen as opposed to in-person to make things clearer. It’s (…) another good skill to have” (*VP1).

Despite the contrast in communication skills developed, volunteer participants felt online volunteering was a *“really different but really valuable experience”* (VP2) and *“requires you to be very prepared for any sort of issue such as connection dropping [or] multiple kids speaking at once”* (VP2). Delivering sessions online allowed volunteers to practice *“speaking to a large group”* and learn *“how to address certain phrases or questions online, because it is so different”* in a range of audiences from *“formal to small group to large audience”* (VP3 and VP9).

Additionally, teacher participants noted how pupils were less engaged during online sessions. This was in part due the loss of “*hands-on involvement*” (*TP2*) as a method of learning, which instead “*felt a lot more like we’re telling you this*” which made it *“hard for them to maintain concentration*” *(TP4)*. This lack of concentration and engagement from pupils made the delivery of some sessions more challenging for volunteers, driving them to develop new ways of communicating with children online.

Medical student volunteer participants felt that both online and in-person prepared them for their paediatric placements by offering exposure to children and opportunities to explain medical concepts to them. However, this experience was attained faster in-person as interactions with children are more direct and “*you appreciate all their different personalities much more*” (VP3).

(iii). *Community and Social*

Volunteer participants described a strong sense of community in-person as you are “*a group of 20 volunteers all wearing the TBH T-shirt*” (VP4) with a shared purpose. In contrast, for “*online sessions there were lots of people I didn’t know as we hadn’t met, so it makes it difficult to deliver quality sessions*” (VP4). Perhaps because online, *“allows people to hide behind their cameras”* (VP3).

Online sessions also made it more difficult for “*basic things such as talking over each other which is just natural in-person (…) with online it was overly formal with stuff like ‘oh no you go first*’” (VP1). Many volunteer participants did not consider meeting online as “*properly meeting*” (VP11) and this may have been due to the lack of social opportunities for example, “*between sessions where you get to talk to people*”, “*talking to your co-presenter*” and “*talking to people whilst setting-up*” which were readily available in in-person sessions. This also could have been due to working online preventing “*other people’s personalities (…) com[ing] through as much*” (VP1) and limiting how much conversation you could have beyond introductions (VP4).

Finally, despite the convenience of not having a commute, some volunteer participants valued “*interacting during the (…) commute [which] added to the sense of community*” (VP1) and felt “*travelling to sessions (…) offered a lot more opportunities to socialise*” (VP10).

Nevertheless, some volunteer participants felt that “*the sense of achievement of doing something as a team carries through online*” as you are “*working together collaboratively for something and this isn’t affected by being online (…) it’s just coming from a different angle*” (VP1).

*“online volunteering does improve your mental wellbeing during and after sessions (…) because it is still socialising (…) which is still important and a contrast to [life in the pandemic]”* (VP11)*.*

In the context of the various lockdowns and government restrictions going on at the time, volunteer participants felt that online volunteering allowed you to “*interact with someone you don’t know (…) which is something I kind of missed during lockdowns*” (VP2).

*“Online volunteering was where I made new friends … [because] obviously you couldn’t talk to that many people during the COVID years”* (VP10)*.*

(iv). *Emotional Wellbeing and Enjoyment*

Volunteer participants emphasised that the play-based, in-person, small-group teaching enabled more personalised teaching and therefore both a unique experience for pupils and a more enjoyable experience for themselves.

*“being able to like use like physical stuff to interact with them (…) I think that’s what really makes TBH so special.” (VP3).*

Lockdowns drastically changed the nature of the university experience; in this context volunteer participants noted the benefits to their mental health of partaking in online volunteering.

*“From the whole situation that we were going through in lockdown (…) I think mentally it was one of the things that kept me sane in it (…) I had a moment where I felt that I was doing something good for other people, not related to academics”* (VP11)*.*

Volunteer participants enjoyed *“seeing the kids (…) [who] have lots of energy (…) and it was nice to feed off that when it was locked down as you weren’t seeing many people”* (VP7)*.* Online also offered a space to *“de-stress (…) [by] working with kids (…) which takes your mind off (…) other issues”* (VP11)*.*

Nevertheless, university teaching was also transferred online, meaning that ICSM-TBH shifting online was yet “*another stare-at-screen session*” (VP10). In-person volunteering offers a “*good [opportunity] to get out of the house*” whereas online sessions could “*feel very weird in the sense that you do it from your own room, as opposed to physically going to the session and getting excited about speaking to all those 5-6-year-olds*” (VP4).

(xxii) *Workload*

The pandemic changed the nature of work for university students; working from home was often “*mentally stressful*”. Online sessions were a *“really fun experience”* and a *“nice kind of break from uni”* (VP12), however sometimes it could be *“a bit challenging to find motivation to join”*, as *“you had been in front of your computer for the entire morning and will be for the entire afternoon for the last five days. So, everything feels like effort and difficult to enjoy”* (VP6). In-person sessions allowed volunteers to “*split up [their] day a bit more as you have to go in-person instead of everything being online*” (VP1).

Convenience was a crucial benefit highlighted in online volunteering as it is “*less time intensive*” and “*easier to fit into schedules*” due to not having travel time and making it “*easier to commit to sessions*” (VP12). In-person volunteering required travelling “*which can impede how many people can turn up*” and can force volunteers to rush from lectures or clinical placements which can be physically and mentally exhausting. Online platforms allowed volunteers to multitask and do things like “*eat lunch during sessions*” or “*do your laundry whilst it is going on*” whereas in-person “*often took place over lunch times*” which disrupted eating schedules for participants (VP10).

## Discussion

This study explored volunteer and teacher perspectives on the value of online versus in-person volunteering with ICSM-TBH. Overall, both online and in-person volunteering showed social and wellbeing benefits and offered opportunities to develop a variety of professional skills for volunteers. However, for volunteers who experienced both formats, in-person sessions were preferred. Volunteers and teachers agreed that in-person sessions provided a more effective learning experience for the pupils. In the context of the national lockdowns and government restrictions, this study demonstrated that online volunteering was an overall positive experience, as it offered a space for socialisation, allowed volunteers to gain a sense of fulfilment and provided a convenient and safe option to volunteer. This study is particularly interesting as it demonstrates that many benefits of volunteering [14] can be obtained online and outside the clinical context. Both modalities were perceived to offer skill development and offer benefits for volunteers’ future professions, especially those interested in paediatrics as a speciality.

The opportunity to socialise through online volunteering was essential, particularly at a time where there were limited social interactions and poor mental health [21]. Online volunteering is helpful when there are no viable alternatives, for example, due to lockdowns or to engage with children who are at high medical risk [22]. Furthermore, whilst to the authors’ knowledge there have been no studies on the impact of online community non-clinical volunteering on student wellbeing during the pandemic, previous studies have highlighted that volunteering in hospital settings during the pandemic increased university students’ sense of wellbeing [14]. This is, in part, due to the feeling of being helpful during the COVID-19 pandemic [14] – a very similar finding to this study. Volunteering may also have supported students in feeling as though they belonged to a community, which is particularly important at times of social isolation. Nevertheless, volunteers did suggest that this sense of enjoyment and community was greater in-person than online. Furthermore, only a subset of ICSM-TBH volunteers took part in this research; these may be volunteers who are highly engaged with the society and therefore demonstrate a stronger sense of enjoyment and community than less committed volunteers.

An important consideration is the impact of screentime on volunteers’ health and wellbeing. During the pandemic, most teaching was online, and overall screentime increased. The introduction of online volunteering further added to the already heightened screentime, which in excess has been associated with worsened wellbeing and increased stress [23, 24]. Therefore, the social benefits of online volunteering need to be considered on balance with possible harms of continued emphasis on remote connectedness – especially once in-person socialising restrictions are lifted.

Although online sessions provided a way for volunteering and teaching to continue during the pandemic, they limited the interaction and engagement with children through lack of props, toys and physical activity. For example, Lourenço et al. describe the importance of educational environments promoting children’s right to play and explored the negative impact of COVID-19 restrictions in Portugal on this [25]. Similarly, Velde et al. demonstrated a significant reduction in activity levels of children during government lockdowns in the Netherlands [26]. Future online volunteering attempts could integrate use of toys and physical activity to improve children’s engagement. However, where in-person volunteering is feasible and safe, it has been demonstrated to be of greater benefit and preferred by both volunteers and teachers, and therefore should be prioritised. Volunteers also described the ability to multitask as a benefit of online volunteering. However, multi-tasking may have reduced volunteers’ engagement with sessions and interactions with children or other volunteers by distracting them and lowering the standard of delivery when compared to in-person. Nevertheless, with the possibility of future public health restrictions in mind and the widespread use of remote teaching and consultations, it is prudent to trial, test, and develop methods to support online learning and volunteering which prioritise interaction and engagement.

Participants also emphasised the potential for personal and professional development across both modalities. Improvement in communication skills and confidence interacting with children were often mentioned as benefits of participating in ICSM-TBH sessions. This strengthens existing evidence by Kis et al., who found that communicating with children in a volunteer setting increased medical students’ comfort when communicating medical information [5]. Whilst both in-person and online sessions were found to promote development of communication skills amongst volunteers, in-person sessions were deemed better for the development of confidence interacting with children, as this was more challenging to maintain their engagement online. Effective communication is important both online and in-person but requires the development of different skillsets. Acquiring both skills is key moving forwards for all medical students, with evidence suggesting that some online elements will be kept in the future of healthcare work, for example remote care in General Practice [27, 28]. Therefore, there are some unique skills acquisition opportunities offered only by online volunteering. Most ICSM-TBH volunteers are in earlier years of medical school; it would be valuable to understand how medical students who have had to practice remote consulting as part of their placement would perceive this difference, especially with increasing body of literature exploring methods to support medical students in developing confidence in this new skill [29, 30].

TBH is a global initiative; despite the organisation of sessions being locally led, the aims of the programme remain constant. Given previous reports of similar benefits of TBH for both students and children, as well as the common goals this initiative, it is hoped that findings of these studies may be helpful in re-designing and adapting TBH and other similar community volunteering programmes in the context of restricted social interaction.

### Limitations

The survey participation rate was approximately one third of all eligible ICSM-TBH volunteers; a larger sample would ensure responses are more representative of volunteer experiences. The survey did not explicitly ask volunteers who participated in both online and in-person sessions about their views on the latter; instead, it only asked for views of online volunteering and a comparison of both. Direct questioning on their views of in-person volunteering might have yielded additional insights. Whilst interviews enabled volunteers’ views to be explored in greater depth, it is possible that those who chose to participate in interviews have greater ties to ICSM-TBH. Furthermore, participants who only volunteered in-person are all in the upper years of medical school, whereas most of those who volunteered online were in more junior years. This may have impacted their perception of the value of ICSM-TBH in-person and online, particularly with regards to professional values and establishment of a community, since more senior students have more clinical experience, and are more familiar with the university environment. Similarly, only teachers who hosted online ICSM-TBH sessions were interviewed, and it is unknown why others declined. It is also important to acknowledge that ICSM-TBH operates in North-West London – perspectives in other areas may yield additional insight into the challenges and individuals disadvantaged by online learning.

## Conclusion

Overall, online volunteering was a positive, beneficial experience for medical students during the COVID-19 pandemic but was not a long-term replacement for in-person volunteering. Both formats were enjoyable experiences for volunteers and children enabling socialisation and community formation, although this was more prominent in-person. A novel skill only gained by volunteers online was the adaptation of communication skills to engage children in a virtual context. Online volunteering was more convenient for volunteers as it saved time and enabled students to multitask, although multitasking may reduce volunteer engagement during sessions. Whilst online volunteering offered an opportunity for social connection during the pandemic and was a chance to continue educating primary school children about healthcare safely, in the absence of major public health and safety concerns, in-person volunteering is preferred by both teachers and volunteers.

## Data Availability

The datasets used and/or analysed during the current study are available from the corresponding author on reasonable request.
